# TFE3-Rearranged Tumors of the Kidney: An Emerging Conundrum

**DOI:** 10.3390/cancers16193396

**Published:** 2024-10-04

**Authors:** Anna Caliò, Stefano Marletta, Matteo Brunelli, Pietro Antonini, Filippo Maria Martelli, Lisa Marcolini, Lavinia Stefanizzi, Guido Martignoni

**Affiliations:** 1 Department of Diagnostics and Public Health, Section of Pathology, University of Verona, 37134 Verona, Italy; anna.calio@univr.it (A.C.); stefano.marletta@univr.it (S.M.); matteo.brunelli@univr.it (M.B.); p.antonini@outlook.it (P.A.); filippomartelli95@gmail.com (F.M.M.); 2 Division of Pathology, Humanitas Istituto Clinico Catanese, 95045 Catania, Italy; 3 Department of Pathology, Pederzoli Hospital, 37019 Peschiera del Garda, Italy; lmarcolini@ospedalepederzoli.it (L.M.); lavinia.stefanizzi@ospedalepederzoli.it (L.S.)

**Keywords:** *TFE3* gene, genetic fusions, renal cell carcinoma, renal PEComa, tumor classification

## Abstract

**Simple Summary:**

The characterization of molecular alterations is continuously gaining relevance in pathology as it can contribute to explaining tumors’ pathogenesis and address specific targeted therapies. As for the kidney, in recent years, much scientific research has been focused on the *MiTF* family genes, particularly on the *TFE3* gene. In this setting, while initially just accustomed to a subtype of renal cell carcinoma, currently classified as TFE3-rearranged renal cell carcinoma, *TFE3* fusions have been identified in a few mesenchymal neoplasms of the kidney viewed as PEComas. In this work, we gather the available data regarding the key clinical and pathological features of these TFE3-rearranged renal epithelial and mesenchymal neoplasms. We seek to propose a comprehensive solution regarding their pathogenesis and sort out their classification conundrum.

**Abstract:**

**Background**: Identical translocations involving the *TFE3* gene and various partners have been found in both renal and soft tissue tumors, like alveolar soft part sarcoma (*ASPSCR1*), ossifying fibromyxoid tumor (*PHF1*), epithelioid hemangioendothelioma, and the clear cell stromal tumor of the lung (*YAP1*). **Methods**: Herein, we review in detail the clinicopathologic and molecular data of TFE3-rearranged renal tumors and propose our perspective, which may shed light on this emerging conundrum. **Results**: Among the kidney tumors carrying *TFE3* translocations, most are morphologically heterogeneous carcinomas labeling for the tubular marker PAX8. The others are mesenchymal neoplasms known as PEComas, characterized by epithelioid cells co-expressing smooth muscle actin, cathepsin-K, melanogenesis markers, and sometimes melanin pigment deposition. Over the past 30 years, numerous *TFE3* fusion partners have been identified, with *ASPL/ASPSCR1*, *PRCC*, *SFPQ/PSF*, and *NONO* being the most frequent. **Conclusions**: It is not well understood why similar gene fusions can give rise to renal tumors with different morpho-immunophenotypes, which may contribute to the recent disagreement regarding their classification. However, as these two entities, respectively, epithelial and mesenchymal in nature, are widely recognized by the pathology community and their clinicopathologic features well established, we overall believe it is still better to retain the names TFE3-rearranged renal cell carcinoma and TFE3-rearranged PEComa.

## 1. Introduction

The *TFE3* gene, located on chromosome Xp11.23, encodes a transcription factor belonging to the Microphthalmia transcription family (MiTF), also including *MiTF*, *TFEB*, and *TFEC*. Identical translocations involving the *TFE3* gene and various partner genes have been found in both renal and soft tissue tumors, such as alveolar soft part sarcoma (*ASPSCR1* gene), ossifying fibromyxoid tumor (*PHF1* gene), epithelioid hemangioendothelioma, and clear cell stromal tumor of the lung (*YAP1* gene).

Among kidney tumors with TFE3 gene translocations, most are morphologically heterogeneous carcinomas positive for the immunohistochemical nuclear tubular marker PAX8. Others are mesenchymal neoplasms known as PEComas, characterized by the presence of epithelioid cells co-expressing smooth muscle actin, cathepsin-K, melanogenesis markers, and sometimes melanin pigment deposition.

Over the past 30 years, numerous *TFE3* fusion partners have been identified, with *ASPL/ASPSCR1*, *PRCC*, *SFPQ/PSF*, and *NONO* being the most frequent. Interestingly, the latter two genes encode proteins that function together as a dimer, regulating many important functions in the cell nucleus, including the coordination of long non-coding RNA molecules into nuclear bodies, speckles, and paraspeckles.

The reason why similar gene fusions can give rise to renal tumors with different morpho-immunophenotypes is not well understood, which may contribute to the recent disagreements regarding their classification.

In this review, we analyzed the clinicopathologic and molecular data of TFE3-rearranged renal tumors reported in the English literature, proposing our perspective on the findings that could help resolve this emerging conundrum.

## 2. *TFE3* Gene

**Figure 1:**
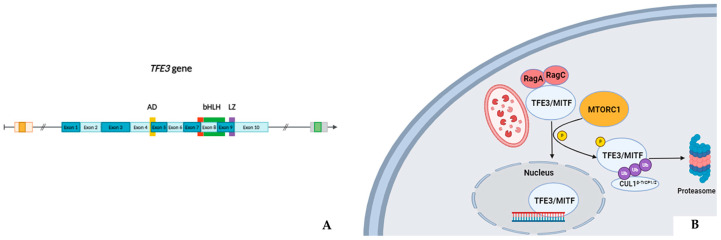
Structure of the TFE3 gene (**A**). Physiologically, the TFE3 transcriptional factor works as a dimer with other MiTF/TFE family proteins. In nutrient-replete settings, these complexes are recruited to the lysosomal membranes by amino acids and RagGTases such as RagA and RagC, which allow MTORC1-mediated phosphorylation and, consequently, ubiquitination by CUL1^β-TrCP1/2^ and proteasomal degradation. On the other hand, the lack of nutrients in starvation/hypoxic conditions lets TFE3 translocate to the cell nucleus, where it can modulate the transcription of key downstream genes involved in lysosomal activity, autophagy, and other catabolic processes (**B**). AD: activation domain; bHLH: basic helix–loop–helix domain; LZ: leucine zipper domain.

The *TFE3* gene, mapped at chromosome Xp11.23, belongs to the MiTF/TFE family, which gathers four different genes: *MiTF*, *TFE3*, *TFEB*, and *TFE3C* [1]. All these genes encode transcriptional factors characterized by a basic helix–loop–helix (bHLH) leucine zipper dimerization domain (Figure 1A) [2]. This latter domain lets them bind specific DNA sequences in the target genes, activating the transcription of molecules involved in key biological pathways, such as TGF-β signaling and downstream factors. The MiTF factor, traditionally the family’s first studied member, plays a pivotal role in melanocytes’ differentiation and function [3]. Similarly, the TFE3 and TFEB proteins share close homology with MiTF and are critically involved in biological processes like osteoclast development [4], allergic diseases [5], and antibody-mediated T-cell immune response [6]. Physiologically, the TFE3/TFEB proteins are located in the cytoplasm, where they are regulated by several enzymatic complexes, including Mammalian Target of Rapamycin Complex 1 (MTORC1), Mitogen-Activated Protein Kinase (MAPK), Extracellular signal-Regulated Kinase (ERK), and Glycogen Synthase Kinase 3 (GSK3). In conditions of nutrient availability, RagGTPases -mediated TFE3/TFEB recruitment to lysosomal membranes by amino acids allows MTORC1 to phosphorylate specific Degron motifs of such transcriptional factors [7]. This keeps TFE3/TFEB from translocating to the nucleus [2] by CUL1^β-TrCP1/2^ chaperone proteins ubiquitination and, ultimately, degradation. Conversely, in hypoxic or starvation settings, the decreased MTORC1 phosphorylation levels let TFE3/TFEB move to cells’ nuclei and prompt the transcription of the critical catabolic regulators involved, such as autophagy and lysosomal activity (Figure 1B) [8].

Genetic alterations of the *TFE3* gene are implied in several pathological conditions. Specifically, missense mutations of some *TFE3* hotspot regions have been linked to a recently described neurodevelopmental syndrome associated with pigmentation defects [9,10]. These mutations have been shown to compromise the TFE3 protein’s ability to bind with RagGTPases and, therefore, constitutively stabilize it and reduce the degradation of this pathogenic form of the protein [7]. Furthermore, translocations involving the *TFE3* gene with several different partners have been described both in renal and soft tissue neoplasms. From a molecular point of view, most of the derived oncogenic fusion proteins are formed of the C-terminal portion of the TFE3 and the N-terminal portion of the related partner. Thus, due to its localization in the N-terminal portion of the *TFE3* gene, during the translocation event, the region encoding the Degron domain is almost always lost, establishing highly stable oncogenic proteins [7]. In these tumors, the resulting over-expressed TFE3 fusion proteins act as aberrant transcription factors activating the expression of multiple downstream targets. Namely, the activation of some molecules normally modulated by the related *MiTF* gene, like cathepsin K or melanocytic markers HMB45 and MART1, explains why immunohistochemical staining for these latter is employed as a useful diagnostic hint [11]. As far as the genetic alternations are concerned, in some tumors, the *TFE3* gene constantly shows specific translocations: among these, the *ASPL/ASPSCR1::TFE3* rearrangement in alveolar soft part sarcoma [12], the *YAP1::TFE3* fusion in clear cell stromal tumor of the lung [13] and a subset of epithelioid angiomyolipoma [14], and the *PHF1::TFE3* translocation in ossifying fibromyxoid tumor [15].

Among TFE3-rearranged tumors arising in the kidney, most have been viewed as morphologically heterogeneous carcinomas staining for the tubular marker PAX8, whereas some others as mesenchymal neoplasms named PEComas. These latter mesenchymal tumors are made up of epithelioid cells, sometimes showing melanin pigment deposition, co-expressing smooth muscle actin, cathepsin K, and melanogenesis markers [16]. Although over the past 30 years, numerous fusion partners have been identified in TFE3-rearranged renal cell carcinoma and PEComa, *ASPL/ASPSCR1*, *PRCC*, *SFPQ*, *NONO*, and *MED15* remain the most frequent in both groups of neoplasms. Several biological functions have been accustomed to these genes in their native and fused forms, playing pivotal roles in tumorigenesis:

- Autophagy/cellular senescence: The *ASPL/ASPSCR1* gene, mapped at 17q25.3, encodes a protein containing a UBX domain which physiologically interacts with glucose transporter type 4 (GLUT4). This protein is a tether, which sequesters the GLUT4 in intracellular vesicles in muscle and fat cells in the absence of insulin and redistributes the transporter to the plasma membrane under insulin stimulation [17]. Experimental models have demonstrated that the aberrant ASPL/ASPSCR1-TFE3 protein induces p21 transcription in a p53-independent manner. This latter further up-regulates pro-inflammatory cytokines associated with autophagy [18] and a senescence-associated secretory phenotype (SASP) [19] which can, ultimately, promote a tumorigenic microenvironment. The *PRCC* gene instead, located at 1q23.1, codifies for a protein playing a role in pre-mRNA splicing. Similarly, the PRCC-TFE3 fusion products have been shown to stimulate the macroautophagy elimination of superfluous, aged, or damaged mitochondria, a process also called “mitophagy”. In detail, the aberrant nuclear PRCC-TFE3 proteins constitutively activate the E3 ubiquitin ligase PRKN which allows cell survival and proliferation under mitochondrial oxidative damage through a reduction in mitochondrial ROS formation [20].
- Speckle–paraspeckles structures: The *SFPQ/PSF* and *NONO* genes, mapped at 1p34 and Xq13.1, respectively, belong to the “Drosophila behavior human splicing” family. They encode for non-coding RNAs playing an essential role in the formation and integrity of the so-called speckle–paraspeckles structures [21]. These membranelles’ nuclear bodies carry out specific regulating functions on the transcriptional process and, overall, gene expression [22] and innate immune response [23]. The proteins they codify work as (hetero)dimers and interact with the NEAT1 long non-coding RNA in constructing the structural backbone of paraspeckles. Notably, plenty of other genes whose fusions are mainly linked to TFEB-rearranged renal cell carcinomas, such as *NEAT1* and *MALAT1*, are involved in forming the speckle–paraspeckles complex. In this view, it is indeed worth mentioning that *SFPQ/PFS::TFE3*-rearranged renal cell carcinoma may morphologically mimic TFEB-rearranged renal cell carcinoma. Such a finding is due to the presence of clusters of small cells or “pseudorosettes” giving various examples of these neoplasms a distinctive biphasic growth pattern [24], more commonly described in TFEB-rearranged renal cell carcinoma [25].
- Lipid accumulation: The *MED15* gene is located at 22q11.21 and its derived protein works as a transcriptional coactivator in RNA polymerase II transcription. Concerning renal cell carcinoma, preclinical models have shown that HIF2α-dependent MED15 over-expression may promote lipid deposition and tumor progression through sterol regulatory element binding protein (SREBP) coactivation via the AKT pathway [26].

These considerations notwithstanding, the underlying reasons why the same gene fusion can give rise to renal tumors with different morphologies and immunophenotypes are not completely understood. Trying to shed light on the solution to this emerging conundrum, we will review in detail the clinical, pathological, and molecular data of TFE3-rearranged renal cell carcinomas and TFE3-rearranged renal PEComa.

## 3. TFE3-Rearranged Renal Cell Carcinoma

**Figure 2:**
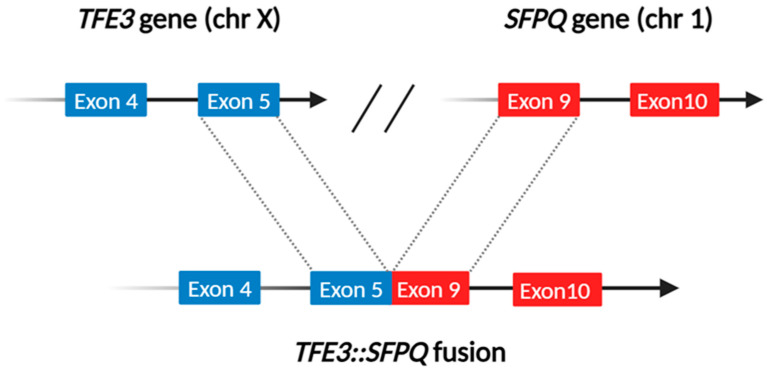
Schematic representation of a *TFE3::SFPQ* fusion, with the breakpoints occurring at exon 5 of the *TFE3* gene (mapped at chromosome 1) and exon 9 of the *SFPQ* gene (located at chromosome 1), respectively.

In the nineties, a *TFE3* gene translocation was reported in a subgroup of papillary renal cell carcinomas [27,28]. However, the first description of TFE3-rearranged renal cell carcinoma is commonly considered in 2001 by Argani who reported eight renal tumors arising in children characterized by a t(X;17)(p11.2;q25) translocation, which fuses the *ASPL/ASPSCR1* and *TFE3* genes [29]. Interestingly, the t(X;17) translocation is also present in alveolar soft part sarcoma, forming a similar *ASPL/ASPSCR1-TFE3* fusion gene. However, the translocation is balanced in TFE3-rearranged renal cell carcinoma and unbalanced in alveolar soft part sarcoma, likely accounting for their clinical and morphological differences [30]. The function of the chimeric TFE3 fusion proteins may also vary, potentially explaining the diverse histologic features observed in this type of renal cell carcinoma. Since then, several translocations involving the *TFE3* gene located on Xp11 have been documented. In addition to the aforementioned t(X;17)(p11.2;q25), the most common translocations in decreasing order of frequency are t(X;1)(p11.2;q21), which fuses the *PRCC* and *TFE3* genes; the t(X;1)(p11.2;p34) [31], which fuses the *SFPQ (PSF)* and *TFE3* genes [32] (Figure 2); inv(X)(p11.2; q12) which fuses *NONO* and the *TFE3* [32]; inv(X)(p11.2; p11.3) which fuses *RBM10* and *TFE3* [33]; and t(X;22) (p11.2; q11.21) which fuses *MED15* and *TFE3* [34].

**Figure 3:**
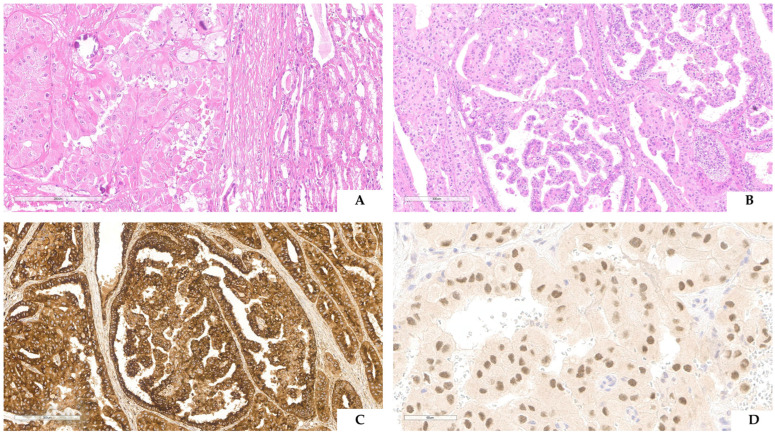
TFE3-rearranged renal cell carcinoma morphological variability, immunohistochemical and molecular findings. A case showing solid–trabecular neoplasms made up of large eosinophilic cells intermingled with several psammoma bodies (**A**). Another one revealed a papillary architecture (**B**), strongly and diffusely labeling for cathepsin K (**C**) and PAX8 (**D**).

Although initially reported in childhood, these tumors can occur in elderly adults. Currently, the average age of onset is approximately 40 years which can be partially explained because the early literature primarily described pediatric cases [35,36]. These tumors, unlike the more common renal cell carcinomas, exhibit a slight female predominance [37]. This may be partially attributed to women’s higher likelihood of X-chromosome rearrangements. The outcome can vary from indolent to rapidly aggressive. Compared with the more common renal cell carcinomas, TFE3-rearranged renal cell carcinoma showed higher stage and frequent lymph node metastases, and, as we previously reported [38], recurrences or metastases occurred within 24 months from surgical resection. It is interesting to note that the type of *TFE3* fusion partner may impact prognosis. For instance, patients with *TFE3::ASPL/ASPSCR1* fusion seem to have a worse prognosis, while patients with *TFE3::MED15* fusion seem to have an excellent prognosis [24]. The several *TFE3* fusion partners may also explain the different morphologies seen in TFE3-rearranged renal cell carcinomas. The most distinctive histologic feature is the presence of papillary architecture composed of large clear cells with prominent nucleoli and frequent psammoma bodies [36]. However, since the initial description, various patterns have been observed, including solid, nested, trabecular, and microcystic architecture [39,40,41] (Figure 3A,B). Papillary architecture is more commonly seen when *PRCC-TFE3* fusion occurs [42], while a multilocular cystic pattern is typically associated with *MED15-TFE3* fusion [43].

Due to the morphologic heterogeneity, several neoplasms, particularly those with clear cells and papillary architecture, can be mistaken for TFE3-rearranged renal cell carcinoma. In this context, immunohistochemistry is a crucial diagnostic tool. Notably, cathepsin K is expressed in approximately 60% of TFE3-rearranged renal cell carcinomas and is consistently negative in other common renal cell neoplasms [44] (Figure 3C). Additionally, melanogenesis markers such as Melan-A and HMB45 are found in TFE3-rearranged renal cell carcinoma, although less frequently compared to their occurrence in TFEB-rearranged renal cell carcinoma. The morphologic heterogeneity and the overlapping immunophenotype are shared with pure epithelioid PEComa/epithelioid angiomyolipoma [45]. In these challenging cases, PAX8 (positive in TFE3-rearranged renal cell carcinoma and negative in pure epithelioid PEComa, Figure 3D) and CD68 (PG-M1) (negative in TFE3-rearranged renal cell carcinoma and positive in pure epithelioid PEComa) are useful markers to reach the proper diagnosis. Among the commonly used immunohistochemical markers, CA9 and cytokeratin 7 are the most helpful immunostainings for differentiating TFE3-rearranged renal cell carcinoma from common renal cell tumors as they are frequently negative in TFE3-rearranged renal cell carcinoma [46]. Recently, other markers have been suggested for supporting the diagnosis of TFE3-rearranged renal cell carcinoma: TRIM63 detected by RNA in situ hybridization (RNA-ISH) [47] and GPNMB (glycoprotein nonmetastatic B) detected by immunohistochemistry [48]. However, as cathepsin K, GPNMB is expressed in *TSC1/2/MTOR* altered renal tumors (including eosinophilic solid and cystic renal cell carcinoma, and PEComa) [49,50].

**Figure 4:**
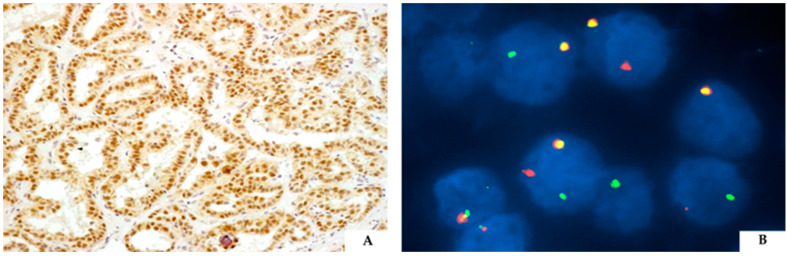
Positive TFE3 immunohistochemical staining in an example of TFE3-rearranged renal cell carcinoma (**A**). FISH in TFE3-rearranged renal cell carcinoma. The distant red and green signals demonstrate the *TFE3* gene translocation using a break-apart probe (**B**).

Nevertheless, since the morphology of TFE3-rearranged renal cell carcinoma is variable and the immunophenotype is not always diagnostic, the diagnosis often necessitates the demonstration of the *TFE3* gene rearrangement. The use of TFE3 immunostaining, regardless of the clones, to demonstrate the translocation is not a reliable surrogate assay due to the not infrequent false-positive and false-negative results [51,52]. Fluorescent in situ hybridization (FISH) is the current gold standard, but it is not always available in laboratories (Figure 4) [53,54,55,56]. Moreover, a univocal cut-off to define a positive case is still lacking and FISH cannot detect subtle TFE3 gene inversions, such as *RMB10* and *NONO* [57,58,59]. In such cases, RNA next-generation sequencing may be able to detect these gene inversions [60,61].

## 4. TFE3-Rearranged Renal PEComa

PEComas are a family of tumors composed of epithelioid cells with clear or eosinophilic cytoplasm frequently with a perivascular distribution (PEC), and co-expressing smooth muscle and melanogenesis markers [62]. This peculiar and almost unique type of cell was initially described in the angiomyolipoma of the kidney and clear cell sugar tumor of the lung [63,64,65]. Since some neoplasms, such as clear cell sugar tumors, were entirely composed of these cells, similar neoplastic elements were looked for and subsequently identified in various sites and diseases, like pulmonary lymphangioleiomyomatosis [66] and angiomyolipoma of the liver [67]. Although these tumors have been reported under different names, including epithelioid angiomyolipoma of the kidney, clear cell sugar tumor of the pancreas, and perivascular epithelioid cell tumor of the uterus and soft tissue, they are now universally recognized as PEComas. In the kidney, the spectrum of PEC lesions includes classic angiomyolipoma (triphasic, leiomyoma-like, lipoma-like) and less common proliferations such as microscopic angiomyolipoma, intraglomerular lesions, angiomyolipoma with epithelial cysts, epithelioid angiomyolipoma, oncocytoma-like angiomyolipoma, and the lymphangioleiomyomatosis of the renal sinus. These lesions can occur in patients with or without Tuberous Sclerosis Complex (TSC) and are driven by mutations in the *TSC1* and *TSC2* genes, which encode for hamartin and tuberin [68,69,70,71]. These proteins form a complex that controls the MTORC1 pathway, which is constantly hyperactivated in these PEC tumors due to the aforementioned mutations. Recently, it has been demonstrated that TFEB is constitutively active in TSC, driving MTORC1 hyperactivation and kidney disease in this syndrome [72,73]. As previously mentioned, *TFEB* is a member of the MiTF/TFE family of transcription factors. It forms homo- or heterodimers with other members of the family and plays a role in melanogenesis as a transcriptional activator of tyrosinase and *TYRP1* genes. Overall, it is considered a master regulator for the transcription of genes involved in lysosome biogenesis and autophagy [74]. Among these transcribed proteins it is certainly to mention cathepsin K, a lysosomal cysteine protease encoded by the *CTSK* gene located on chromosome 1q21.3, which is highly expressed in all PEComas arising in and outside the kidney [16,75].

**Figure 5:**
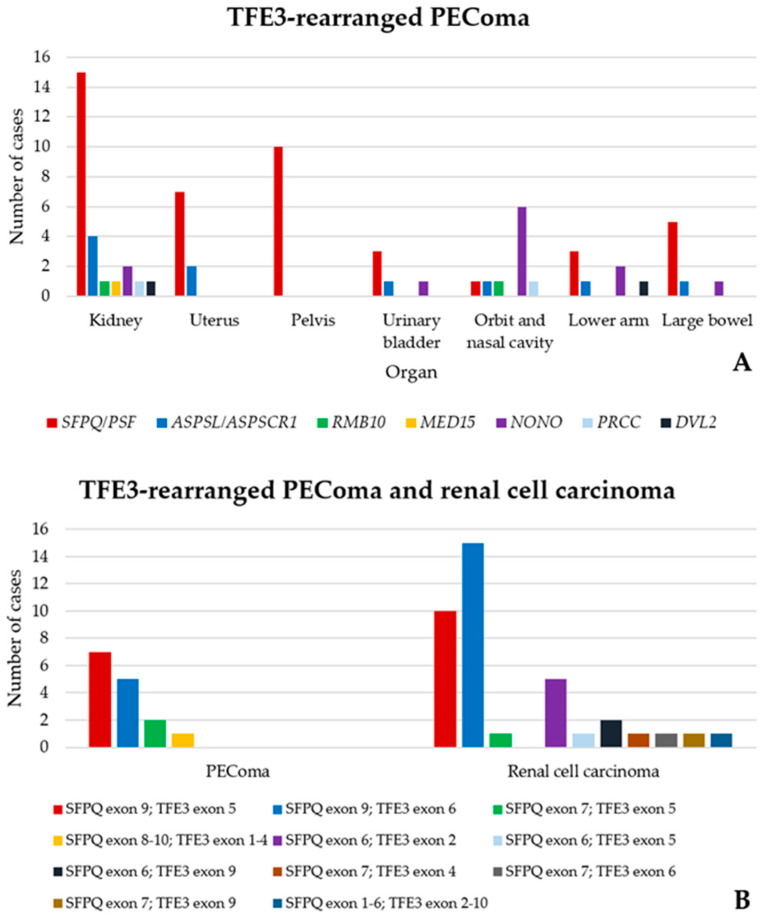
Genetic fusion pattern distribution among TFE3-rearranged PEComas from different sites, highlighting the higher frequency of kidney tumors [39,42,76,77,78,79,80,81,82,83,84] compared to the other organs (**A**). Break-point distribution in SFPQ/PFS-TFE3-rearranged PEComas and renal cell carcinomas (**B**).

In 2009, a subset of PEComas carrying *TFE3* gene fusions, expressing melanogenesis markers, and rarely showing immunoreactivity for muscle markers, was identified in young patients without TSC [76]. Although none of the TFE3-rearranged PEComas in the initial study occurred in the kidney, 35 of such tumors have since been found in this organ, making it the most frequent site for this neoplasm [39,42,76,77,78,79,80,81,82,83] (Figure 5A). Renal TFE3-rearranged PEComas show a female predominance (17 females versus 11 males, 60% versus 40%) and occur in young patients (10 cases in the second decade, 37%). Six out of 14 patients died of the disease, most after developing metastases. Regarding molecular rearrangements, among the neoplasms with available data, the *SFPQ/PSF::TFE3* fusion was the most frequent genetic alteration found (14 cases, 74%) (Figure 5B) [79], followed by *ASPL/ASPSCR1::TFE3* (3 cases, 16%) [78], *RBM10::TFE3* (1 case, 5%) [83], and *MED15:TFE3* (1 case, 5%) [84]. Compared to TFE3-rearranged PEComas occurring in other sites (especially the uterus, bladder, and soft tissues), those in the kidney arise less frequently in females (60% versus 73%), in younger patients (second versus fourth decade of life), and less frequently carry the *NONO::TFE3* rearrangement, which is instead the second most common alteration (17%) behind *SFPQ/PSF::TFE3* in extrarenal TFE3-rearranged PEComas [85].

**Figure 6:**
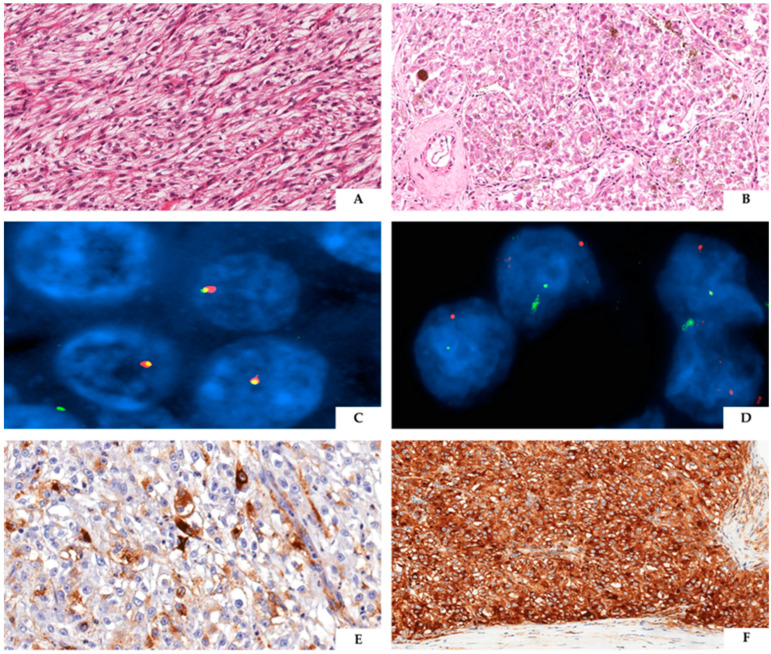
Conventional PEComa composed of spindle cells arranged in a fascicular pattern (**A**). TFE3-rearranged PEComa made up of clear to eosinophilic cells displaying a nested to solid carcinoma architecture, not uncommonly with melanin pigment deposition (**B**). FISH images from conventional PEComa (**C**) and TFE3-rearranged PEComa (**D**), the latter revealing multiple breaks of the investigated probes witnessing an underlying genetic fusion. As for immunohistochemistry, TFE3-rearranged PEComas usually stain positive for melanocytic markers, like HMB45 (**E**), and cathepsin K (**F**).

The immunohistochemical analysis of renal TFE3-rearranged PEComas consistently shows a robust expression of cathepsin K (99%) and melanogenesis markers (HMB45 91% and MART1/MELAN A 80%) along with negativity for PAX8, cytokeratins, and S100 (Figure 6). Regarding muscle markers, only occasional positivity for smooth muscle actin has been described (19%), while desmin was negative in all the evaluated cases [86]. Therefore, the immunoprofile of renal and extrarenal TFE3-rearranged PEComas overlaps, but it slightly differs from PEComas related to *TSC1/TSC2* mutations, in which the expression of muscle markers, particularly actin, is more frequently observed. Furthermore, melanin pigment deposits have been identified in about half of renal TFE3-rearranged PEComas. Namely, while some of these tumors showed numerous heavily pigmented cells, others just contained small amounts of melanin. The presence of melanin pigment in some renal TFE3-rearranged PEComas has been considered a distinctive feature from PEComas carrying *TSC1/2* mutations, leading some authors to propose reclassifying them as “melanotic Xp11 neoplasms” [84].

## 5. Emerging Conundrum

In the previous paragraphs, we have summarized the main data from the literature regarding the characteristics of the *TFE3* gene and its translocations, highlighting the primary functions of the fusion partners and, whenever possible, the genes and fusion proteins derived from these translocations. We have also described the main features of renal neoplasms carrying these translocations, known as TFE3-rearranged renal cell carcinoma and renal TFE3-rearranged PEComas. These considerations notwithstanding, several questions arise: (a) Is it relevant to distinguish carcinomas and mesenchymal tumors in the context of neoplasms carrying the same TFE3 gene translocation? (b) How can identical gene fusions drive tumorigenesis but result in neoplasms with different morphology and immunophenotype? (c) Is there any point in currently classifying mesenchymal renal neoplasms carrying *TFE3* rearrangements as PEComas?

The answer to the first question depends on the significance we assign to the data discussed in the previous paragraphs. Basically, a tumor’s essence is defined either by its histogenesis—the cell from which it is believed to originate—or by the molecular events driving its neoplastic evolution. Therefore, regarding the TFE3-rearranged tumors originating in the kidney, which is more important, the translocation involving the *TFE3* gene itself or the differentiation lineage of the cells it occurs in? Currently, the latter model is more widely acknowledged. Namely, if the *TFE3* translocation occurs in a renal tubular stem cell, the neoplastic transformation will produce a renal cell carcinoma expressing PAX8 and CD10 (i.e., markers positive in the proximal tubule of the kidney). Conversely, if the same translocation verifies in a mesenchymal stem cell, it will cause the onset of a PEComa. When comparing the biological behavior of tumors classified as TFE3-rearranged renal cell carcinoma with neoplasms diagnosed as TFE3-rearranged PEComa, both show aggressiveness and young age at the time of identification, with the former appearing more aggressive and arising in younger patients. Therefore, these characteristics seem to support their classification into two distinct entities based on histogenesis.

While we recognize that it is unclear whether all the same gene fusions described in TFE3-rearranged renal cell carcinoma and TFE3-rearranged PEComa are truly identical, one possible explanation for why the same gene fusions can drive tumorigenesis and result in neoplasms with differing morphology and immunophenotype is that the effects of the same translocation may vary depending on the cell type. For example, the occurrence of an *SFPQ::TFE3* translocation in a PAX8-positive renal tubular stem cell will give rise to renal cell carcinoma, which has a 78%, 38%, and 44% chance of staining for CD10, cathepsin K, and HMB45, respectively. In contrast, these chances are 0%, 100%, and 91% in a PEComa originating from PAX8-negative mesenchymal stem cells. The different percentages of the expression of the melanogenesis marker HMB45 explain why melanin pigment is identified more frequently in renal TFE3-rearranged PEComas (80%) than in TFE3-rearranged renal cell carcinoma of the kidney (33%). Another issue that has not yet been sufficiently investigated is the relevance of the type and biological activity of the fusion partner involved in the *TFE3* rearrangement. This problem has been partially addressed among TFE3-rearranged renal cell carcinomas using an unsupervised transcriptomic analysis, which identified and characterized five molecular subtypes [87]. All carcinomas with *ASPL/ASPSCR1::TFE3* fusion were classified into the high angiogenesis/stroma/proliferation cluster, whereas half of the *MED15::TFE3* and *SFPQ/PSF::TFE3* tumors were included in the cluster enriched for EMT, apical junction, TGF-β, WNT catenin, and hypoxia signaling. Finally, the group mainly composed of *NONO::TFE3* and *SFPQ/PSF::TFE3* fusion neoplasms featured a low expression of angiogenesis modules and moderate enrichment of E2F signaling [87]. Regarding TFE3-rearranged PEComas, 74% and 49% of the renal and extrarenal tumors carry *SFPQ/PSF::TFE3* and *NONO::TFE3* rearrangements, respectively. The latter is the second most frequent fusion (17%) in extrarenal TFE3-rearranged PEComas. As previously mentioned, both *SFPQ/PSF* and *NONO* genes are part of the “drosophila behavior human splicing” family, which codes for proteins that, as heterodimers, interact with the NEAT1 long non-coding RNA to construct speckle–paraspeckle structures [21,22,23]. These genes’ products have also been documented to be involved in several other key cellular processes, such as cell cycle progression, DNA damage repair, the regulation of telomere homeostasis, and spliceosome assembly among others [88]. To note, due to their plenty (and yet to be fully discovered) role, NONO and SFPQ have been claimed as “transcriptional supermediators” [88]. Additionally, alongside renal cell carcinomas and PEComas, aberrant NONO and SFPQ function was initially described in B-cell leukemia [88] and, later, in many other types of solid tumors, like neuroblastoma [89], melanoma, hepatocellular, colorectal, breast, and prostate cancer [90,91,92,93,94]. Indeed, the exact driving pathogenic basis for each tumor according to the specific *NONO* and *SPFQ* molecular alterations is still unknown. However, based on these considerations, it is tempting to speculate that in renal cell carcinomas and PEComas, the functions of the fusion partner genes *SFPQ/PSF* and *NONO* could play an important role in the stem cell in which they occur and subsequently in the morphogenesis of the arising tumor,

In recent years, there has been debate about whether it is appropriate to classify HMB45-positive mesenchymal neoplasms carrying *TFE3* rearrangement as PEComa [84,86]. In 2020, Wang et al. suggested calling these tumors “melanotic Xp11 neoplasms” instead of “Xp11 translocation PEComa.” To support this change, the Authors took into account the presence of melanin pigment in the neoplastic cells of many of these tumors, their aggressive behavior, and gene expression profiling data. Their unsupervised clustering analysis suggested that melanotic Xp11 neoplasms and alveolar soft part sarcomas form a distinct cluster, clearly different from TSC-related PEComas and TFE3-rearranged renal cell carcinoma. However, those melanotic Xp11 neoplasms arising in the kidney clustered with TFE3-rearranged renal cell carcinoma rather than with alveolar soft part sarcoma. In 2024, Argani et al. also proposed renaming these tumors as “TFE3-rearranged PEComa-like neoplasms”, highlighting the younger age of the patients, the association with prior exposure to chemotherapy, and the almost constant lack of muscle marker expression compared to TSC-related PEComas [78]. Nevertheless, in this study, although they recognized that extensively melanotic pigmented TFE3-rearranged PEComas, originally described as “melanotic Xp11 translocation renal cancer”, exist on the spectrum of these tumors, they avoided including this characteristic in the name. They supported this choice by noting that melanin pigment is much more frequently seen in renal rather than extrarenal TFE3-rearranged PEComas, suggesting the possibility that at least some of them may derive from a different cell of origin. We believe that both proposals have strengths and weaknesses. Wang and colleagues highlighted melanin pigment as a distinctive characteristic between TFE3-rearranged PEComas and TSC-related PEComas, especially in tumors arising in the kidney [84]. However, this difference could be related to the different underlying molecular mechanisms. Namely, a translocation involving the *TFE3* gene, a powerful stimulator of melanogenesis, in TFE3-rearranged PEComas, and the action of TFEB migrating to the nuclear site due to low levels of RagC-GDP, which prevents TFEB recruitment to MTORC1 and its subsequent phosphorylation in TSC-related PEComas [7]. In several other neoplasms, such as melanotic schwannoma, melanotic carcinoid, melanotic PNET, and melanotic dermatofibrosarcoma protuberans, despite melanin pigment being easily identified, the tumor name is not modified by this circumstance. Therefore, we question whether this characteristic is sufficient to change the name of TFE3-rearranged PEComas at this time. Argani et al. also highlighted the absence of muscle marker expression in TFE3-rearranged PEComas as a distinctive characteristic among TSC-related PEComas. However, it is important to remember that PEComas were defined as tumors characterized by the presence of perivascular epithelioid cells (PECs), unique cells able to “modulate its morphology and immunophenotype… [which] can show muscular features with a spindle shape and a stronger positivity for actin than for HMB45 or it can have an epithelioid feature with a strong positivity for HMB45 and a mild, if any, reaction for actin” [62].

For all these reasons, we believe it is better to retain the name TFE3-rearranged PEComa, which accurately identifies its clinical, pathological, and molecular characteristics and is well-known by clinicians and pathologists worldwide.

Finally, as for the next future directions, further research at the biological level may deepen our understanding of renal TFE3-rearranged tumors. For example, single-cell transcriptomic analysis could help define cellular origins, identify key drivers and mechanisms of tumorigenesis, and offer therapeutic insights. Spatial transcriptomic analysis could also uncover valuable data, such as gene expression signatures in tumors like TFE3-rearranged renal cell carcinoma and PEComa, which exhibit tumor heterogeneity [95]. Both intra-tumor and inter-tumor heterogeneity are well known to affect prognosis and treatment response across cancer types significantly [96]. Accurate diagnosis, along with the identification of molecular signatures that predict the most effective therapies, would offer substantial clinical benefits. The expression of GPNMB in TFE3-rearranged tumors could represent a potential therapeutic target. Clinical trials have shown some anti-tumor activity using novel antibody-drug conjugates targeting GPNMB in various cancers, including breast cancer, melanoma, and osteosarcoma [97,98,99]. Another promising therapeutic target might be cathepsin K. Cabozantinib, a tyrosine kinase receptor inhibitor that targets VEGFR and MET, has been shown to reduce cathepsin K activity, suggesting its potential usefulness in the targeted treatment of cathepsin K-positive TFE3-rearranged renal cell carcinoma [38,100].

## Data Availability

No new data were created or analyzed in this study. Data sharing does not apply to this article.
